# Evaluation of Canine Neonatal Health by Breeders: A Prospective Questionnaire Study on the Association between Neonatal Scores (Modified APGAR), Parturition, Birth Weight, Growth, and Puppy Mortality

**DOI:** 10.3390/ani13233605

**Published:** 2023-11-22

**Authors:** Eva Axnér, Rebecca Axelsson, Ulrika Hermansson

**Affiliations:** 1 Division of Reproduction, Department of Clinical Sciences, Faculty of Veterinary Medicine and Animal Science, Swedish University of Agricultural Sciences, P.O. Box 7054, 750 07 Uppsala, Sweden; rebecca.c.axelsson@gmail.com; 2 University Animal Hospital, Swedish University of Agricultural Sciences, P.O. Box 7054, 750 07 Uppsala, Sweden; ulrika.hermansson@slu.se

**Keywords:** Apgar, dog, inter-pup interval, neonatal, reproduction

## Abstract

**Simple Summary:**

Puppy mortality is a widespread problem in small animal medicine. Apgar scoring, adapted for puppies performed by trained veterinary staff, was previously shown to be useful in identifying newborn puppies with an increased risk of mortality. The majority of puppies, however, are born in a home or kennel environment. In most whelpings, early evaluations and interventions are performed by the breeder. Therefore, we wanted to evaluate if a modified Apgar protocol for neonatal monitoring would be usable by breeders in a home environment. Our aim was to evaluate potential associations between neonatal scores, delivery times, birth weights, growth rates, and puppy mortality. Twenty-one bitches gave birth to 113 puppies by vaginal delivery. Neonatal scores were related to puppy mortality. Puppies with low neonatal scores had a longer expulsion interval and lower viability. Mortality was higher in puppies with a low relative birthweight. Puppies with a negative growth rate the first two days after parturition did not have a significantly higher risk of mortality in this study. Our results indicate that a modified and simplified neonatal scoring, performed by breeders approximately 5 min after birth, could be useful to identify puppies at risk of mortality.

**Abstract:**

Mortality of neonatal puppies is a widespread problem in small animal medicine. Neonatal monitoring, according to standardized protocols, can be useful for identifying puppies that are at risk of mortality. Prompt intervention on weak puppies could increase survival rates. Apgar scoring adapted for puppies has been demonstrated to be associated with mortality and is usually performed by trained veterinary staff. The majority of puppies, however, are born in a home or kennel environment and not at a veterinary clinic. Our aims were, therefore, to evaluate if a modified protocol for neonatal monitoring would be usable by breeders in a home environment. We wanted to evaluate potential associations between modified Apgar scores, birth weights, delivery times, growth rates, and puppy mortality. Modified Apgar scores were related to the viability of live-born puppies (*p* < 0.0013). The viability and expulsion time of each puppy were significantly related (*p* = 0.010 with all puppies included and *p* = 0.038 with only live-born puppies included). Viability was significantly related to relative birthweight (*p* < 0.01). Puppies with a negative growth rate the first two days after parturition did not have a significantly higher risk of mortality. In conclusion, a modified and simplified Apgar scoring performed by breeders approximately 5 min after birth was associated with puppy mortality.

## 1. Introduction

Neonatal mortality is a widespread problem in small animal medicine [1,2,3,4] and is both an emotional and economic matter for breeders. Mortality can be due to several factors, such as prolonged or dystocic parturition [1,5], infectious diseases [1,6], malformations, or lack of appropriate care for newborns [7,8]. The majority of puppy deaths occur in the first two to three weeks of life [1,2,9]. Rates of stillborn puppies vary with breed and studies, with reported averages between 4.3% and 15% [2,3,9,10] and total losses between 9% and 24% [2,10,11]. By the identification of risk factors for puppy mortality, it may be possible to increase survival rates. Prompt intervention during parturition or for neonatal puppies could increase puppy survival.

Different prognostic risk factors for poor neonatal survival have been identified. Puppies with low birth weights were more likely to die within the first 24 h after birth [9,12]. High birth weight was also a risk factor for stillbirth in colonies of guide dogs [5]. A high birth weight might increase the risk of neonatal mortality by increasing the length of parturition [5,13], which in turn could lead to intrapartum asphyxia [1,13,14]. Hypoxemia is one of the leading causes of neonatal deaths [14]. A prolonged second stage and a prolonged interval between births of puppies are associated with lower neonatal survival rates [5,15]. Groppetti et al. [16], evaluating vaginal delivery in 65 puppies, reported that Apgar scoring tended to worsen with prolonged delivery, although not significant.

The growth rate is an indication of the puppy’s ability to ingest colostrum and milk. Breeders commonly evaluate growth rates by weighing puppies daily. Although some degree of weight loss is not uncommon after birth, growth curves should be positive within the first few days [17]. A growth rate of ≤4% after day 2 was associated with a higher risk of neonatal mortality in puppies [18]. Failure to ingest colostrum on the first day of life will not only affect growth but will also have a negative impact on passive immunity [9,19]. By identifying puppies that fail to grow, the breeder may intervene. 

Apgar scoring was first described in human medicine and has been widely used ever since. Veronesi et al. [20] presented a modified Apgar scoring system for the evaluation of neonatal health in puppies and demonstrated that it was associated with puppy mortality. Apgar scoring in humans is performed at one and five minutes after delivery [21] and was originally evaluated five minutes after birth in puppies [20]. Heart rate, respiration, muscle tone, reflex irritability, and membrane mucus color are assessed and assigned a value of 0 to 2 [20,21,22,23,24]. In contrast to humans, bitches give birth to litters with several puppies, and exactly timed scoring may be difficult, depending on the availability of assistants and litter size. Mila et al. [12] showed, however, that Apgar scoring performed within 8 h after birth also was predictive of puppy mortality. Apgar scoring was a better predictor of neonatal survival than blood lactate in both humans and puppies [12,21] and is easy to use with no requirement for special equipment. Apgar scoring of puppies is usually performed by trained veterinary staff [12,16,22]. However, most puppies are born in a home environment or a kennel and are not evaluated by a veterinarian until their first vaccinations. Therefore, the breeder performs most of the early interventions. A systematic evaluation by breeders would thus have an even larger impact on puppy survival than evaluations performed at clinics. 

Our aims were to evaluate a protocol for neonatal evaluation adapted for use by breeders and to identify factors indicating an increased risk for puppy mortality. Therefore, we evaluated potential associations between modified Apgar scores, birth weights, delivery times, growth, and puppy mortality.

## 2. Materials and Methods

Data were collected prospectively. Questionnaires were sent out by e-mail to breeders who had reported an interest in participating in the study. Participating breeders were recruited through advertisements on social media channels and mailings sent out by Royal Canin to their national network of breeders in Sweden. 

Dog breeds included in the study were Affenpinscher (one bitch, three puppies); Belgian Shepherd Dog/Tervueren (one bitch, five puppies); Cane corso (one bitch, eight puppies); Chihuahua, long-haired (one bitch, three puppies); Chinese Crested Dog (one bitch, four puppies); Dachshund miniature, wire-haired (one bitch, six puppies); Danish-Swedish Farmdog (one bitch, five puppies), English Cocker Spaniel (one bitch, five puppies); German Spaniel (two bitches, nine and six puppies); Griffon Fauve de Bretagne (one bitch, six puppies), Icelandic Sheepdog (one bitch, five puppies); Irish Red Setter (two bitches, eight and nine puppies); Maltese (one bitch, four puppies); Norfolk Terrier (one bitch, four puppies); Pomeranian (one bitch, four puppies); Poodle, miniature (one bitch, four puppies); Portuguese Podengo, (Warren Hound) smooth-haired/miniature (one bitch, three puppies); Schipperke (one bitch, four puppies); and Weimaraner, long-haired (one bitch, eight puppies). A total of 19 dog breeders with 21 bitches, giving birth to 21 litters with a total of 113 puppies born by vaginal delivery in Sweden from May to September 2018, were included. Bitches of the same breeds belonged to the same breeders.

### 2.1. Neonatal Scoring

Participating breeders were instructed to evaluate neonatal scores (NS) according to a modified Apgar protocol. According to instructions, puppies were evaluated five minutes after birth. The protocol used in our study was a modified version of the Apgar protocol designed by Veronesi et al. [20] to be usable for breeders. Respiration rate, mucus membrane color, movements, and righting reflex were recorded (Table 1).

### 2.2. Body Weight, Birth Weight, and Growth

Breeders were instructed to record the birth weight of the puppies and their daily weight until day 14. Thereafter, weights were recorded every week until 8 weeks of age. The bitche’s body weight (BW) before pregnancy was recorded; the sire’s BW was also recorded but was not considered further in the analysis because of collinearity with the mothers’ body weights.

### 2.3. Viability

Viability was divided into five categories: (0) puppies that were stillborn, (1) puppies that were born alive but died within 2 h, (2) puppies dying between 2 and 24 h after birth, (3) puppies dying between 24 h and one week, and (4) puppies that were still alive after one week.

### 2.4. Statistics

The relative birth weight of the puppy was calculated as a proportion of the mother’s body weight. The growth rate was calculated as the increase in weight from birth divided by birth weight. The percent daily growth rate was calculated as the increase in weight from the previous day/weight the previous day. Neonatal scores were divided into three categories: scores 0–3, (1) scores 4–6, and (2) scores 7–8 (3). The BW of the mothers was divided into four groups: <10 kg, 10–19.9 kg, 20–30 kg, and >40 kg.

On a puppy level, Fisher’s exact test was used to evaluate NS in relation to the viability of live-born puppies and to evaluate the risk of mortality in relation to a negative growth rate. 

A mixed model was used with relative birth weight as the outcome (logarithmic transformation) and with litter as a random factor and gender, the BWgroup of mother, and birth order as fixed factors. A mixed model was also used to evaluate relative growth after 7 weeks (log10 of BW /birthweight and BW/mother BW) in relation to adult the BWgroup and gender, with litter as a random factor. The goodness-of-fit was tested by plotting residuals vs. fitted data and by checking the normal distribution of the residuals.

Ordinal regression was used with viability as an outcome and expulsion time (start of the stage to birth for firstborn puppies, interpup interval for subsequent puppies), birth order, and a group of relative birth weight as fixed factors. The ordinal regression was calculated both for all born puppies, including stillborn, and for only live-born puppies. The relative birth weight was divided into two groups: (1) the lowest quartile of each BW group of mothers and (2) puppies from quartiles 2–4. 

The effect of relative birth weight on expulsion time for each puppy was evaluated with the non-parametric Kruskal–Wallis test because normal distribution could not be achieved. 

On a litter level, Spearman’s correlation was used to evaluate the relation between litter size and maternal BW, and the mean and total birthweight/litter, total length of parturition, mean relative birth weight, and the total relative birth weight/litter. Statistical analyses were made with Minitab 21.4.1 (© 2023 Minitab, LLC, https://www.minitab.com, accessed on 1 September, 2023) and in R (R Core Team, 2022) [25]. Litter size, sire´s BW, and bitch´s body weight were highly correlated. Therefore, these parameters were not included as factors in the same models to avoid collinearity [26].

## 3. Results

### 3.1. Descriptive Statistics and Correlations

Pregnancy lengths, calculated from first mating, ranged between 58 and 65 days, and maternal age between 2 and 8.5 years. A total of 10 out of 21 bitches were primiparous (47.6%). Median litter size was 5.0 (range 3 to 9) puppies. No medication was used at the time of delivery except for one bitch to stimulate contractions after signs of placental detachment; the medication used was not specified. 

The sex ratio was 55.7% females (63 females and 50 males). Five puppies were stillborn (4.4%). Four out of five stillborn puppies belonged to the same litter. Nine puppies were born alive but died within the first week, resulting in a total neonatal puppy mortality of 12.4%. After the first week, only two more puppies died, one unexpectedly on day 12 and one Pomeranian puppy that was euthanized after three weeks due to suspected hydrocephalus. This was the only reported congenital defect in this study (0.9%). The distribution of viability and Apgar scoring are presented in Table 2.

### 3.2. Parturition

**Figure 1:**
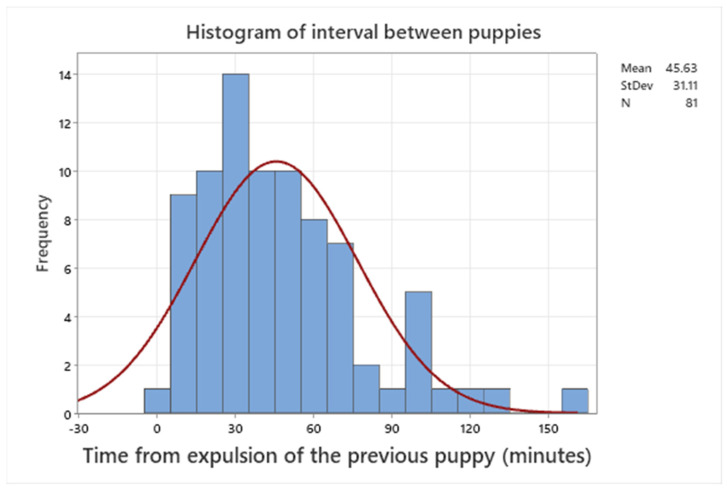
Histogram of the inter-pup interval of puppies with viability 4 (alive after more than one week).

The median interval between the observed start of the second stage and the expulsion of the first puppy was 46 min (range 0–900 min). Three puppies were reported to have been born 0 min after the beginning of stage 2. Excluding these three, the range was 6–900 min with a median of 48.5 min. For live-born puppies, the range was 0–250 min. The median time interval between expulsions of each puppy was 41.5 min (range 0 to 285) minutes. For live-born puppies, the median interval between puppies was 40.0 min (range 0 to 165 min). The time in stage 2 for each puppy was not affected by relative birth weight (*p* > 0.05). Puppies that were still alive after more than one week after birth were born with a median of 40 min after the previous puppy (Figure 1).

The median total length of parturition, calculated as the time from the observed onset of stage II until delivery of the last puppy in each litter, was 285 min (range 30 to 1545 min). The total length of parturition was significantly and positively associated with litter size (*p* < 0.001, Rho = 0.79). 

### 3.3. Neonatal Scores, Viability Birth Weight, and Litter Size

**Figure 2:**
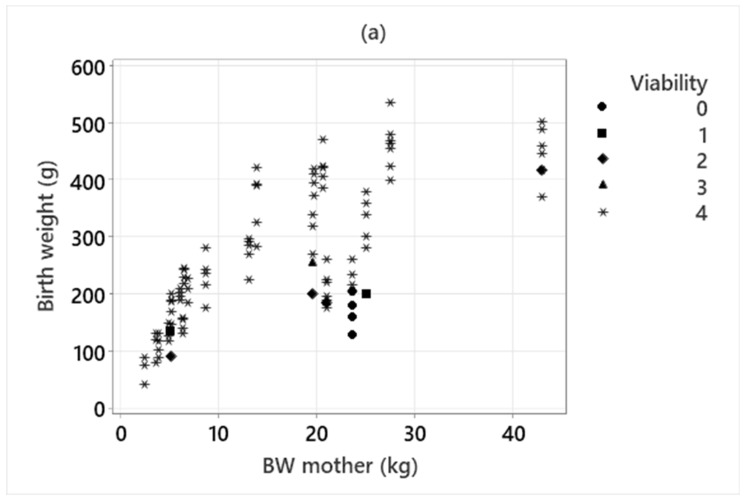

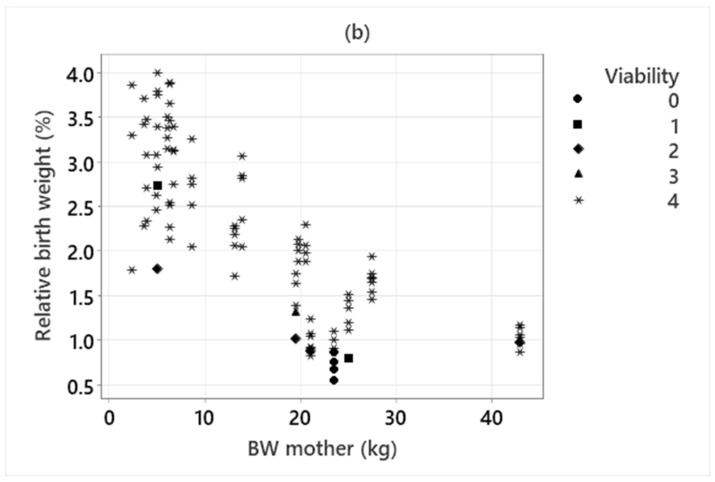
(**a**) Birth weight plotted against maternal body weight. (**b**) Relative birth weight plotted against maternal body weight. Relative birth weight is calculated as (puppies’ weight at birth/maternal body weight) × 100. Viability is indicated with different symbols. 0 = stillborn, 1 = live-born but died < 2 h after birth; 2 = died 2–24 h after birth; 3 = died between 24 h to one week after birth; 4 = alive for more than one week.

Birth weight ranged between 45 and 535 g. Relative birth weight ranged between 0.55 and 4.0% (Table 3). The mean and total birth weight in the litter were significantly correlated with the body weight of the mother (*p* < 0.001, Rho = 0.84 and *p* < 0.001, Rho = 0.96 respectively) (Figure 2a). The mean relative birth weight in the litter was also significantly correlated with the body size of the mother, with larger mothers giving birth to relatively smaller puppies (*p* < 0.001, Rho = −0.83) (Figure 2b). However, the total relative birth weight of the litters was not significantly related to the mother’s body weight (*p* = 0.76, Rho = 0.24) and ranged between 6.8 and 18.7% of the mother’s body weight. Litter size was significantly correlated with the mother’s BW (*p* < 0.001, Rho = 0.86). Puppy gender had a significant effect on relative birth weight (*p* = 0.001), with males being heavier than females. Birth order did not affect relative birth weight (*p* = 0.18).

The neonatal score group was related to viability (*p* = 0.0013), indicating that the NS could predict post-partum mortality (Table 2). Viability was significantly affected by the expulsion time of each puppy (*p* = 0.010 and *p* = 0.038 for all puppies and only live-born, respectively). Puppies in the lowest quartile of relative birth weight had lower viability both with all puppies (*p* < 0.001, OR 45.6), as well as with only live-born puppies included in the model (*p* = 0.002, OR 22.5) (Figure 2b). Birth order did not affect viability (*p* > 0.05). Of 28 puppies in the lowest quartile, 11 (39.3%) died before the first week.

### 3.4. Growth

**Figure 3:**
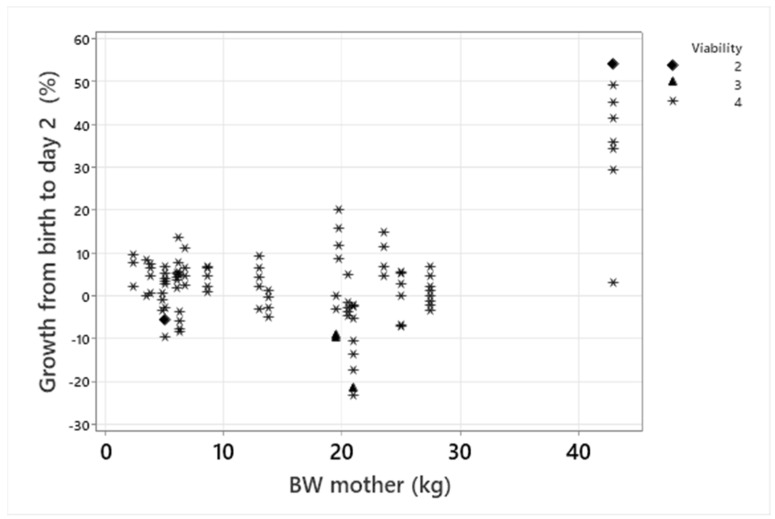
Relative growth of puppies the day after birth plotted against maternal BW the day after birth. Viability is indicated with different symbols. % growth = ((weight day 2-birthweight)/birthweight)) * 100.

**Figure 4:**
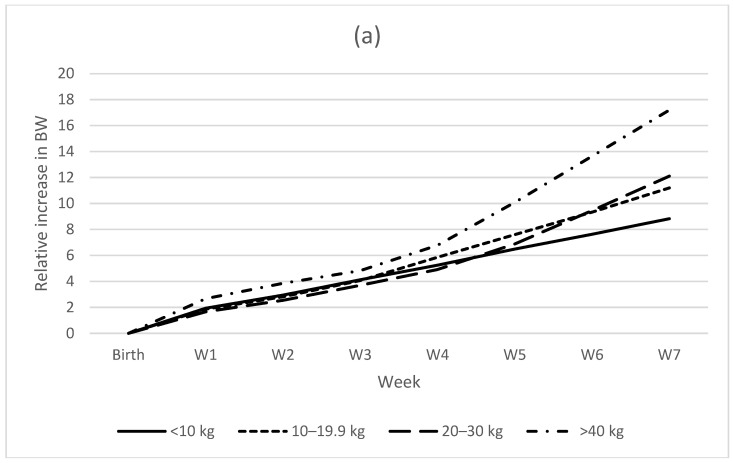

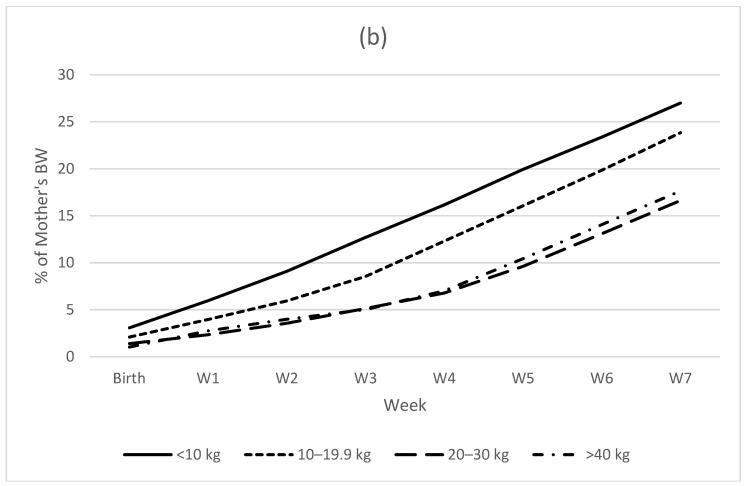
(**a**) Weekly relative growth of puppies (BW/birth weight) according to the mothers’ BW group. (**b**) Weekly growth in relation to the mother’s BW (body weight/mother’s BW). Mean values for each week.

Growth rate in the first 24 h ranged between −23.1% and 54.6% with a median of 2.9%. Of all puppies with weight recorded the day after birth, 40 decreased or did not change weight from birth, while 63 increased in weight. Of 34 puppies that had decreased in weight the day after birth, three died by the first week of age (Figure 3). The risk of mortality for puppies with negative growth on day 2 was not significant (*p* = 0.10). Thirteen puppies had not increased in weight two days after birth, of which none died within the first week, and 12 survived until weaning. One week after birth, the puppies had, on average, almost doubled their birth weight (mean BW/birthweight = 1.88, range 1.08–3.09, Figure 4a).

Seven weeks after birth, the puppies were, on average, 10.9 (range 4.97–21.29) times heavier compared with their birth weight (Figure 4a). Relative growth varied according to the mother’s BW group, with a larger relative increase in the 7th week in larger breeds (*p* < 0.006) but with no effect of gender (*p* = 0.21). In contrast, the puppies’ relative BW in relation to the mother’s BW was still higher for smaller breeds at 7 weeks of age (*p* < 0.007, range 9.79–35.3, Figure 4b) and higher for males (*p* < 0.001).

## 4. Discussion

### 4.1. Neonatal Scoring, Parturition, and Viability

According to our data, standardized neonatal surveillance according to a modified Apgar scoring can be performed by breeders. There was a significant relationship between the NS and the viability of live-born puppies. This indicates that the scores were useful in identifying puppies that were at higher risk of mortality. It remains to be elucidated if such puppies can be rescued with more intense monitoring. It cannot be ruled out that the instructions for monitoring the puppies could have contributed to a higher puppy survival because of closer observation of the neonates. The stillbirth rate in our study (4.4%) was, however, in the lower range of previous studies, with 3.4–10.9% of puppies being stillborn [2,9,18,27]. Total mortality (14.1%) was similar to another study, with 13.4% of all puppies dying the first two months after birth [27] but also in the lower range compared with some other studies [1,3].

Scoring performed by breeders may give a larger variation compared with scoring performed by trained veterinary staff. We used a simplified Apgar score to standardize the breeders’ evaluations of neonatal scores. Instructions were made as simple as possible to reduce operator-dependent variation. In order to simplify the protocol, scoring of heart rate was excluded, as this parameter is difficult to evaluate for a nonprofessional. A reduced heart rate in neonatal puppies is closely related to asphyxia [1]. Therefore, it is likely that heart rate, respiratory rate, and mucus membrane color are all parameters related to oxygenation. Standardized observation of reflexes, mucus membranes, respiration rate, and movements is feasible for breeders. Puppies that survived the first 24 h had a high probability of surviving until weaning at 8 weeks of age. This confirms results from previous studies stating that the risk of mortality is highest in the early neonatal period. Our data confirms that a prolonged parturition process has a negative impact on neonatal health [5,15]. Stillbirth, as well as viability in live-born puppies, were significantly affected by the time of stage 2 in each puppy. Only two puppies with viability four were born more than 2 h after the previous littermate (Figure 1). According to the literature, dystocia should be suspected if there are more than 2–4 h between births of puppies [28,29,30]. Although live and vigorous puppies can be born several hours after a previous puppy, our data confirms that most healthy puppies are born within 2 h after the previous littermate. An interval exceeding this time is associated with increased mortality [5].

### 4.2. Birth Weight

The absolute and relative birth weights of the puppies in this study were within ranges previously reported for breeds of similar sizes [31,32]. As previously reported, we found a negative relationship between adult size and relative birth weight [31,32]. Although the absolute birth weight increased with the size of the mother, larger mothers gave birth to relatively smaller puppies. While the median individual relative birth weight in the litter was related to the mother’s body weight, in contrast to Schrank et al. [31], this was not the case for the total relative weight of the litter. This was most likely because larger mothers, although giving birth to relatively smaller puppies, also gave birth to larger litters. We found that males had a higher relative birth weight than females, which is in accordance with previous studies [5,33,34,35], although not consistently [18,31]. 

Previous studies have demonstrated that puppies with a low birth weight have a higher probability of neonatal mortality [18,31]. Low birth weight, as well as threshold values, have been defined somewhat differently in different studies [1,18,36]. Because of the large variation in maternal weight in our study, we chose to use relative birth weight rather than absolute birth weight as a factor in the statistical evaluations. The relative birth weight was significantly associated with viability, with puppies in the lowest quartile having a lower viability. Of puppies in the lowest quartile, 39% were stillborn or died within the first week after birth. It remains to be elucidated if threshold values for puppies at risk should be set according to individual maternal body weight, breed [33,36], or breed group [18]. Considering that larger breeds normally give birth to relatively smaller puppies, both relative and absolute birth weights should probably be evaluated according to expected adult body weight. 

In contrast to a previous study [5], there was no relationship between relatively larger puppies and viability in our data. Neither was there a relationship between relative birth weight and inter-pup interval, indicating that larger puppies did not have a more difficult birth. This is in contrast to some other studies indicating that larger puppies may be at a disadvantage by causing a more difficult and prolonged birth [5,28]. A puppy weight of 4–5% of the weight of the bitch is considered to be associated with an increased risk for birth complications [28]. The highest relative birth weight in this study was 4% in two puppies with two different mothers, both weighing 5 kg. Both of these had expulsion times within normal limits (15 min after the last puppy and 46 min after the beginning of stage 2). The low proportion of relatively large puppies in this study might also be a reason for the lack of a relationship between relatively larger birth weights, parturition time, and viability.

### 4.3. Growth

The median growth rate on the day after birth was 2.9%. Although three of four puppies with known growth rates that died in the first 24 h had a decrease in body weight after birth, the majority of the puppies with negative growth on the first day of life survived until weaning. Puppies with a negative early growth rate in the first 2 days of life may be at a higher risk for mortality [18], but it has also been reported previously that the majority of puppies with an early negative growth will survive [18]. Growth rate and mortality were not significantly related in our study. A very low rate of mortality after 24 h might contribute to the lack of effect of growth rate in this study. Some degree of weight loss in the first days after birth has been considered to be normal and related to loss of urine and meconium [17], while other authors report that most puppies increase in weight in the first two days of life [4]. On day 4, four puppies from two litters still had a lower body weight than their birth weight. In both of these litters, there was the mortality of littermates, and a possible pathology was found (cardiac murmur) in one of the three puppies surviving until weaning. On the fourth day of life, the growth curve should probably normally be positive with total recovery of birth weight [17]. Median growth rate, in relation to birthweight, the first week after birth was 88% in our study, which is similar to a study on large breeds (84%) [9] and higher than the mean growth rate in a study including breeds of different sizes (67.1%) [4].

## 5. Conclusions

In conclusion, our data suggest that instructing breeders to perform a modified and simplified neonatal scoring approximately 5 min after birth could be of value for systematic surveillance of neonatal puppy health in a home environment. Data collected by breeders are likely not as precise as data collected by scientists. However, the protocols were simplified to standardize the data as much as possible. The modified Apgar scores and relative birth weights were related to puppy survival. Puppies with lower Apgar scores had significantly longer inter-pup intervals during parturition. By instructing breeders to routinely evaluate breathing, mucous membranes, reflexes, and vocalization, more at-risk puppies can be detected. It remains to be elucidated if systematic neonatal monitoring by breeders can also contribute to reducing puppy mortality.

## Figures and Tables

**Table 1:** Scoring system used in this study.

	Score for Each Parameter		
	0	1	2
Respiration	<6 breaths/min	6–12 breaths/min	>15 breaths/min
Mucus membranes	Cyanotic	Pale	Pink
Movements	Absent	Weak	Normal, active
Reflexes	No turning No vocalization	Turns in 5–10 s Weak vocalization	Turns in <5 s Clear vocalization

**Table 2:** Distribution of mortality in live-born puppies in different Neonatal Score (NS) groups.

		Viability Categories *			
NS	Puppies n	1	2	3	4
7–8	93	0	4	1	88
4–6	12	0	1	1	10
0–3	3	2	0	0	1

* 1 = live-born but died < 2 h after birth; 2 = died 2–24 h after birth; 3 = died between 24 h to one week after birth; 4 = alive for more than one week.

**Table 3:** Summary of birth weights according to the mother’s BW.

Mother’s BW (Range)	N Litters (N Puppies)	Birth Weight (g) Median (Range)	Relative Birth Weight % Median (Range)
2.3–8.6	11 (44)	173 (41–280)	3.14 (1.78–4.00)
13–19.7	4 (22)	320 (200–423)	2.07 (1.03–3.06)
20.5–27.5	5 (39)	300 (130–535)	1.20 (0.55–2.3)
43 kg	1 (8)	446 (371–502)	1.04 (0.86–2.3)

## Data Availability

Datasets are available from the author upon reasonable request.
